# Global trends in depression among patients living with HIV: A bibliometric analysis

**DOI:** 10.3389/fpsyg.2023.1125300

**Published:** 2023-03-09

**Authors:** Xiaoyu Du, Qian Zhang, Jiaqi Hao, Xilong Gong, Jing Liu, Jia Chen

**Affiliations:** ^1^Xiangya Nursing School, Central South University, Changsha, China; ^2^Xiangya Hospital Department of Neurosurgery, Central South University, Changsha, China; ^3^Department of Breast Disease, Henan Breast Cancer Center, The Affiliated Cancer Hospital of Zhengzhou University and Henan Cancer Hospital, Zhengzhou, China

**Keywords:** depression, human immunodeficiency virus, HIV/AIDS, bibliometric analysis, VOSviewer, CiteSpace

## Abstract

**Background:**

Human immunodeficiency virus (HIV) related depression has seriously affected the quality of life and treatment outcomes of patients living with HIV (PLWH), which has become a hot topic in recent years. This study aims to discover the main keywords, predict frontier topics, and give meaningful suggestions for researchers by bibliometric analysis.

**Methods:**

Publications between 1999 and 2022 on depression in HIV/AIDS were searched in the Web of Science core collection. Microsoft Excel 2010 and VOSviewer were utilized to key contributors (e.g., authors, journals, institutions, and countries). VOSviewer and CiteSpace were used to analyze the knowledge evolution, collaborative maps, hot topics, and keywords trends in this field.

**Results:**

In total, 8,190 publications were included in the final analysis. From 1999 to 2021, the number of published articles roughly presents a steadily increasing trend. The United States, South Africa, and the United Kingdom were three key contributing countries/regions to this field. University Calif San Francisco (United States), University Calif Los Angeles (United States), and Johns Hopkins University (United States) were three key contributing institutions. Safren, Steven A. was the most productive and highest cited author. AIDS Care was the top prolific journal. Antiretroviral therapy and adherence, men has sex with men, mental health, substance abuse, stigma, and Sub-Saharan Africa were the central topics regarding the depression-related research in HIV/AIDS.

**Conclusion:**

This bibliometric analysis reported the publication trend, major contributing countries/regions, institutions, authors, journals and mapped the knowledge network of depression-related research on HIV/AIDS. In this field, topics such as “adherence,” “mental health,” “substance abuse,” “stigma,” “men who have sex with men” and “South Africa” have attracted considerable attention.

## 1. Introduction

Human immunodeficiency virus (HIV) infection and its’ caused acquired *immunodeficiency* syndrome (Organization and Unaids) are rapidly evolving and still is a global public health challenge. HIV/AIDS ranks as the top 10 causes of disease-related life loss in adolescents (9th, 10–24-years old) and adults (2nd, 25–49-year old) in 2019, a significant increase compared to 1999 (13th, 25–49-year old; Abbafati et al., 2020). The Joint United Nations Programmer on HIV/AIDS (Organization and Unaids) has reported that 38.4 million people are living with HIV in 2021, with 1.5 million new infections and 650 thousand deaths due to HIV-related causes (World Health Organization, 2021). Since the widespread promotion of antiretroviral therapy (ART), a large percentage of patients living with HIV (PLWH) have a lifespan comparable to that of uninfected individuals (Kirtane et al., 2018). Despite receiving effective ART, PLWH continue to be plagued by residual immune dysregulation, which increases their risk for neuropsychiatric comorbidities (Carrico et al., 2022). *Depression* is one of the most common comorbidities among adult HIV-positive individuals, with prevalence of ~20–40% (Pence et al., 2018). Prior studies have reported that depression in HIV-positive people is 2–3 times higher than that in HIV-negative comparison subjects (Ciesla and Roberts, 2001). Furthermore, PLWH often face the prospect of social stigma, loss of social support, loneliness, and decreased self-esteem (Berger et al., 2001; Armoon et al., 2022). Depression is not only a clinical disorder but also affects the patient’s health outcome. On the one hand, depression affects adherence of ART, on the other hand, it weakens the effectiveness of treatment and even increases the mortality rate of patients (Pence et al., 2018).

Many scholars are devoted to studying the association between HIV and depression. As early as 2001, Ciesla et al. have evaluate the relationship between HIV infection and depression through a meta-analysis (Ciesla and Roberts, 2001). Leah H et al. have conducted a comprehensive review of the link between depression and cognition among PLWH (Rubin and Maki, 2019). Others have reviewed depression in women living with HIV (Fauk et al., 2022), men who have sex with men (MSM; Nouri et al., 2022; Operario et al., 2022), and interventions for improving depression in HIV patients (Ibeneme et al., 2022), etc. However, these conventional review papers only focus on subdomains and analyze limited relevant literature. There is still lacking a complete and macroscopic analysis of the entire field literature.

Bibliometrics is the cross-disciplinary science of quantitative analysis of the keyword clusters and hotspot bursts in a given field by mathematical, bibliographical, and statistical methods (Yao et al., 2013). It typically analyzes the publication trends, institutions, countries, journals, citation of articles, and the most contributing researchers by applying bibliometric software (e.g., Bibliometrix R, Gephi, Pajek, CiteSpace, and VOSviewer). Additionally, it is used to constructed maps to visualize the hotspots and research evolutions (Moretto et al., 2019). Some published bibliometric studies analyzed subdomains of AIDS, such as AIDS medication adherence (Sweileh, 2018; Ye and Zhang, 2019), geriatrics HIV (Hoang et al., 2022), HIV-related dementia (Hussain et al., 2022) HIV-related stigma (Sweileh, 2019). These contribute to an understanding of the specifics of a particular area of HIV. Although Tran et al. conducted a bibliometric analysis on depression among HIV/AIDS in 2019 (Tran et al., 2019), there are some shortcomings that need to be addressed: 1. they did not analyze the top co-citation references, the keywords co-occurrence, and trends of the keywords; 2. they used only one bibliographic analysis tool, VOSviewer, which is quite limited; 3. the search time only included up to 2017. Importantly, after the outbreak of the COVID-19 pandemic, research on depression among PLWH needs to be updated and explored further. Thus, in this study, we analyzed depression-related research on HIV/AIDS using the CiteSpace and VOSviewer (Pan et al., 2018; Zhang et al., 2022a,b,c). This study aims to answer the following research questions.

1. Identify key contributors (e.g., authors, journals, institutions, countries) and how they collaborate.
2. Discover main topics and knowledge evolution based on keywords and co-cited references.
3. Overview this field, predict frontier topics, and give meaningful suggestions for researchers.

## 2. Methods and materials

### 2.1. Search strategy and data collection

On September 06, 2022, we conducted an advanced search on the Web of Science Core Collection (WoSCC). Search formulas were “TS = (depress* OR depression OR depressed OR depressive disorder OR mood disorder)” AND “TS = (HIV OR HIV AND AIDS OR human-immunodeficiency-virus OR acquired immune deficiency syndrome),” to identify publications primarily focused on HIV in the field of depression. The date ranged from 1999/01/01 to 2022/09/06, language type was set as English, and the document type was limited to articles and reviews. First, two authors (Xiaoyu Du and Qian Zhang) independently searched the WoSCC and then screened the title, abstract, or full manuscript to identify whether these publications met this research topic. Second, the other two senior scholars (Jing Liu and Jia Chen) read and evaluated the discrepancies between the former authors, and discussed with them until a consensus was reached. All publications were exported as the format of “Full Record and Cited References,” and then used for bibliometric parameters extraction.

### 2.2. Data analysis

Firstly, we used VOSviewer (version 1.6.11, Leiden University, Leiden, Netherlands) to extract the following bibliometric parameters, mainly including the following elements, e.g., title, keywords, authors, institutions, countries/regions, published journals, publication year, total citations (TC), citations per publication (CPP) and cited references. Then we used Microsoft Excel 2010 (Redmond, Washington, United States) to identify the publication trend, the top contributors (e.g., prolific authors, institutions, and countries, journals). And we mapped the collaboration network between authors, institutions, and countries by VOSviewer, to demonstrate their scientific influence in this field. CiteSpace (Version 6.1. R1) were used to map the co-cited references network and identify references with highly citation burst. Co-occurrence of author keywords and keywords with citation burst were used to visualize the knowledge evolution, hot topics and potential research frontiers in this filed. In the map of VOSviewer and CiteSpace, the node size positively related to the number of publications, and the line width positively associated with their link strength.

## 3. Results

### 3.1. General data

**Figure 1:**
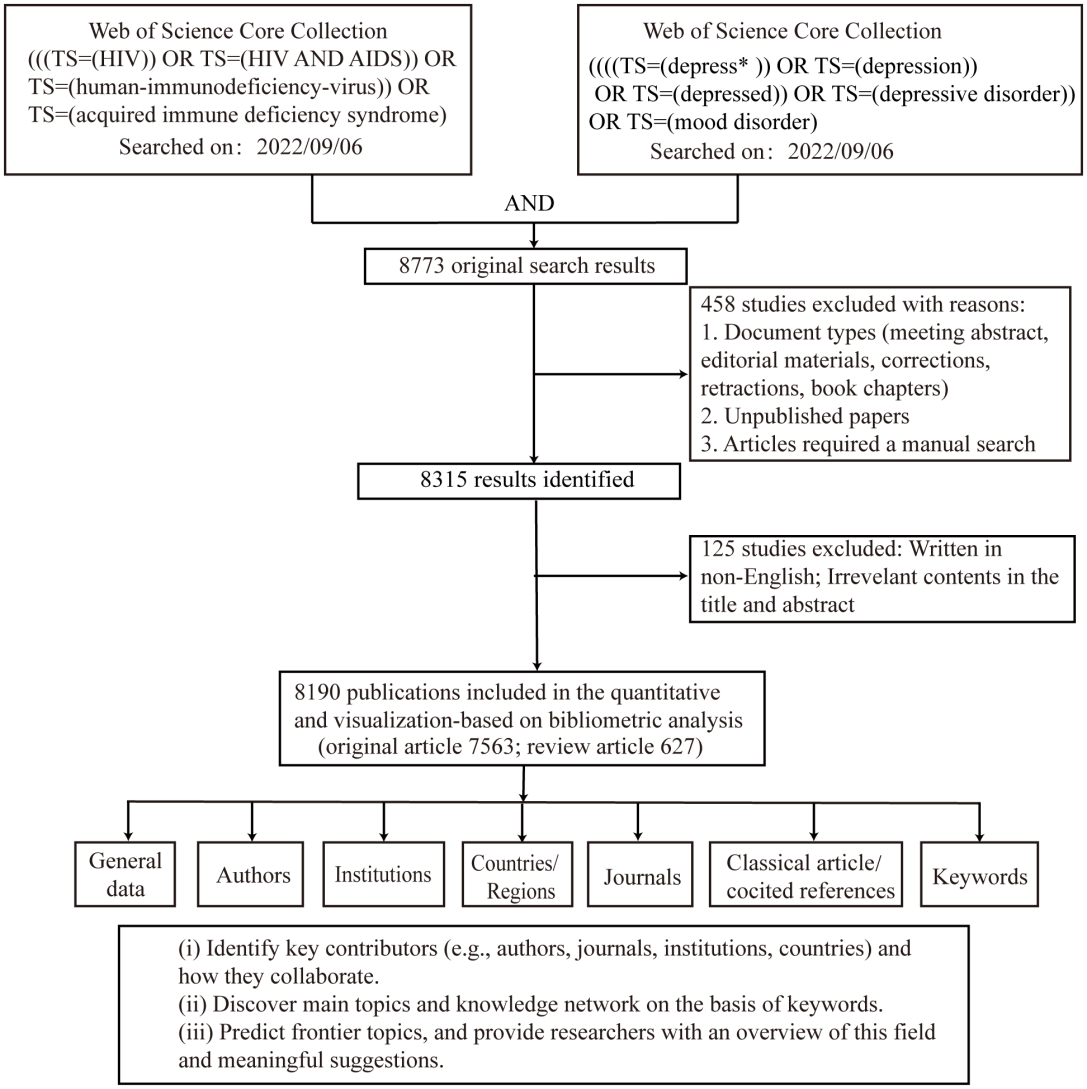
Data screening flow chart. The literature search was performed on WoSCC and language limited to English, and steps of bibliometric analysis.

Figure 1 shows the study design and analytic approach. After restrictions on the document types (original article and review) and the language (English), 8,190 publications were included in the final analysis. In total, 30,026 authors, 6,582 institutions, 140 countries/regions, and 1,308 journals participated in these publications.

### 3.2. Publication trend

**Figure 2:**
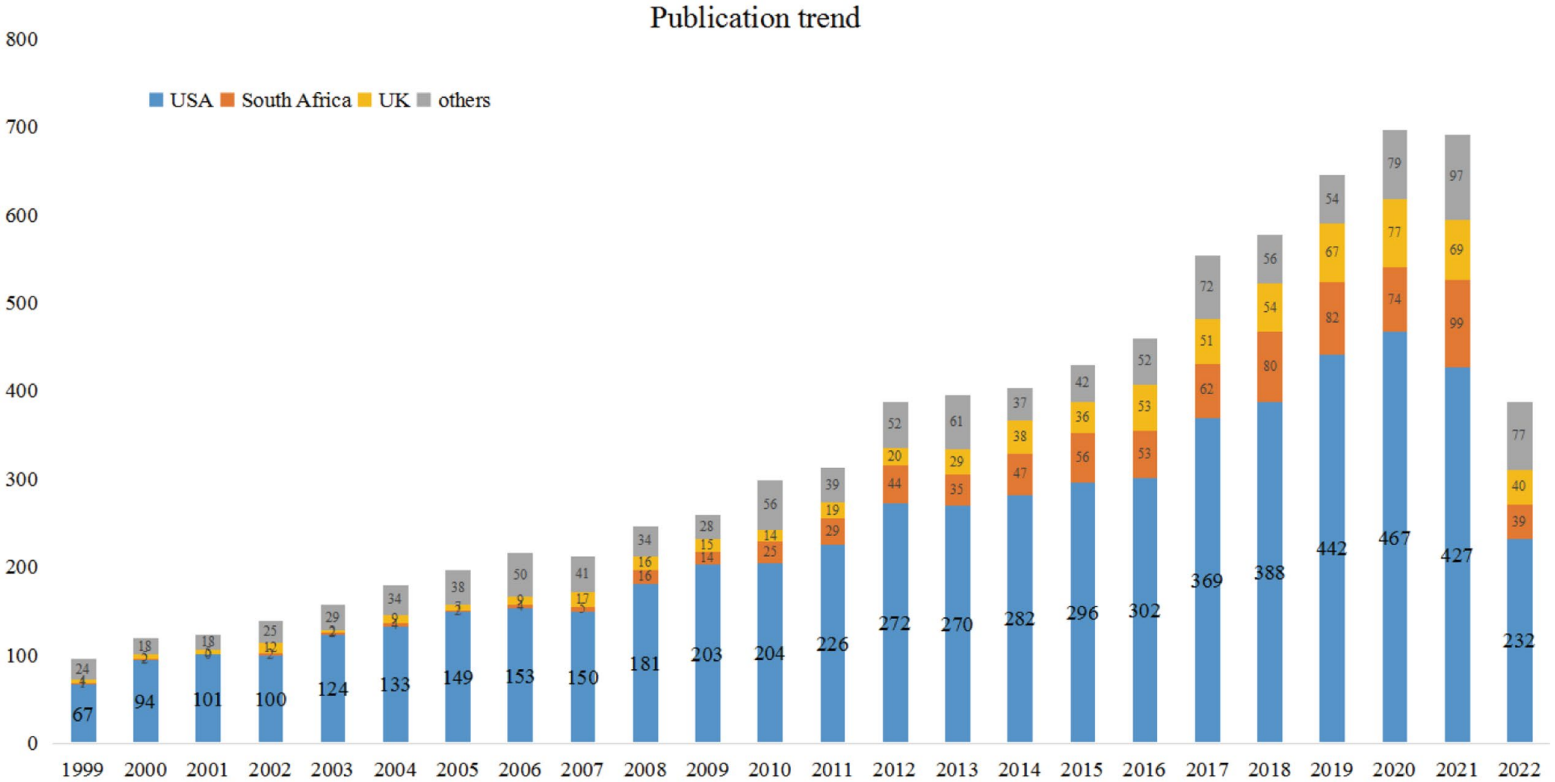
The number of publications each year related to depression in HIV/AIDS.

Figure 2 displays the publication trend. From 1999 to 2021, the number of publications roughly presents a steadily increasing trend, and 388 publications as of the first 9 months in 2022. In addition, we presented the publication trend of the top three prolific countries.

### 3.3. Top contributing countries

**Figure 3:**
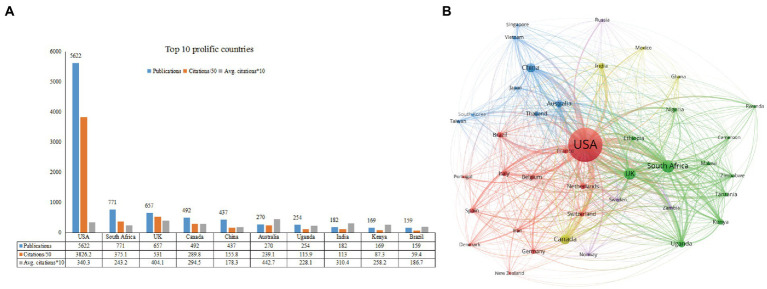
The top 10 most prolific countries and inter-countries co-operation relationships on depression-related research in HIV/AIDS. **(A)** The number of publications, citations/50, and Avg citations*10 in the top 10 countries. **(B)** The co-authorship network visualization map of countries for depression-related research in HIV/AIDS. Node size indicated the number of articles produced. The distance between any two nodes is positively associated with the cooperation strength. The color indicated the cooperation between different clusters of countries.

Figure 3 displays the top 10 most prolific countries and the collaborative network between them. The United States ranked first with 5,622 publications (68.6%) and 191310 TC, followed by South Africa (771 publications, 18755 TC) and the United Kingdom (657 publications, 26550 TC; Figure 3A). As for CPP, Australia ranked first with 44.3 CPP, followed by the United Kingdom (40.4 CPP) and the United States (34.03 CPP). To present the international collaborative network, we utilized the module of co-authorship-country in VOSviewer. With at least 30 publications, a total of 42 prolific countries/regions formed a cooperative network. Of which, the United States, South Africa, the United Kingdom, Canada and China are large nodes with relatively thicker links. The United States had the highest degree of co-operation total link strength (TLS = 45,077) and co-operated with 39 prolific countries. Of which, South Africa (TLS = 15,833), and the United Kingdom (TLS = 13,858) had a closer academic cooperation with the United States.

### 3.4. Top contributing institutions

**Figure 4:**
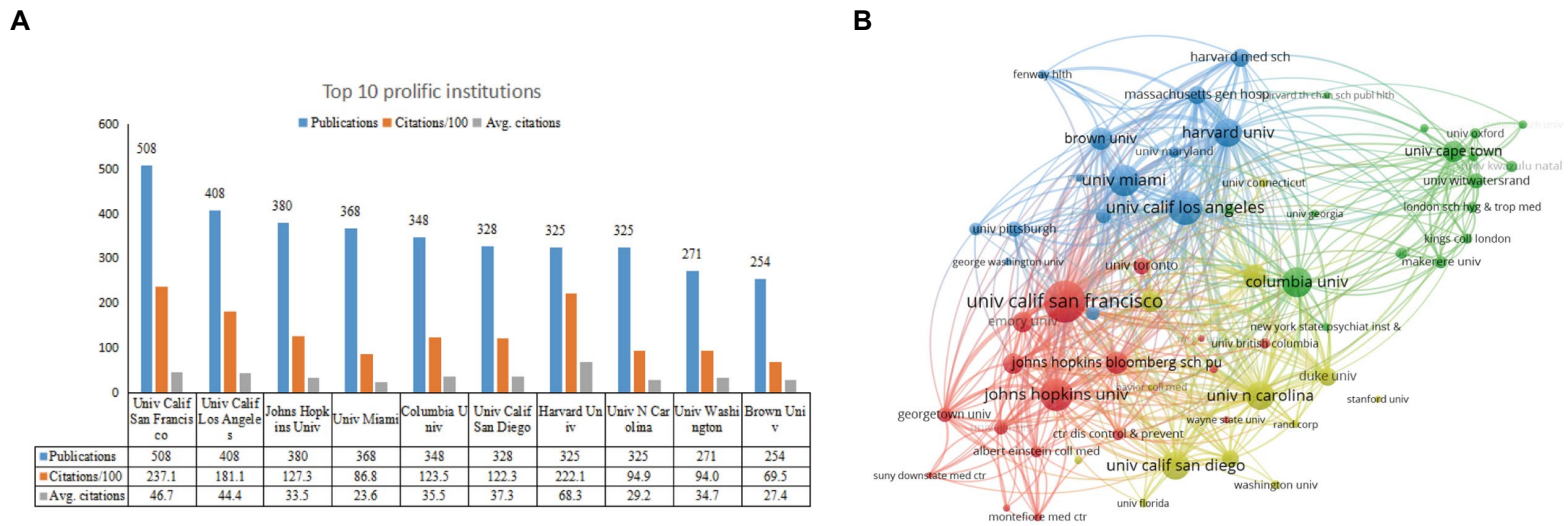
The top 10 most productive institutions and inter-institution co-operation relationships on depression-related research in HIV/AIDS. **(A)** The number of publications, citations/100, and Avg citations in the top 10 institutions. **(B)** The co-authorship network visualization map of the institution for depression-related research in HIV/AIDS. Node size indicated the number of articles produced. The distance between any two nodes is positively associated with the cooperation strength. The color indicated the cooperation between different clusters of institutions.

Figure 4 displays the most prolific institutions which published at least 70 articles. The top 10 prolific institutions published 42.9% of publications. University Calif San Francisco (United States) ranks first with 508 publications, followed by University Calif Los Angeles (United States) with 408 publications, Johns Hopkins University (United States) with 380 publications. In terms of citations, University Calif San Francisco (United States) ranks first with 23709 TC and 46.7 CPP, followed by Harvard University (United States) with 22206 TC and 68.3 CPP, and University Calif Los Angeles (United States) with 18107 TC and 44.4 CPP. We used VOSviewer to visualize the institution collaborative network. As shown in Figure 4B, the top 60 prolific institutions were grouped into four clusters. The biggest cluster was institutions that primarily came from the United States (e.g., University Calif San Francisco, University Calif Los Angeles, Johns Hopkins University, University Alabama Birmingham). The red, blue, and yellow clusters contain mainly United States institutions, and the green cluster contains mainly African institutions. However, United States institutions and African institutions are mainly associated with Columbia University.

### 3.5. Top contributing authors

**Figure 5:**
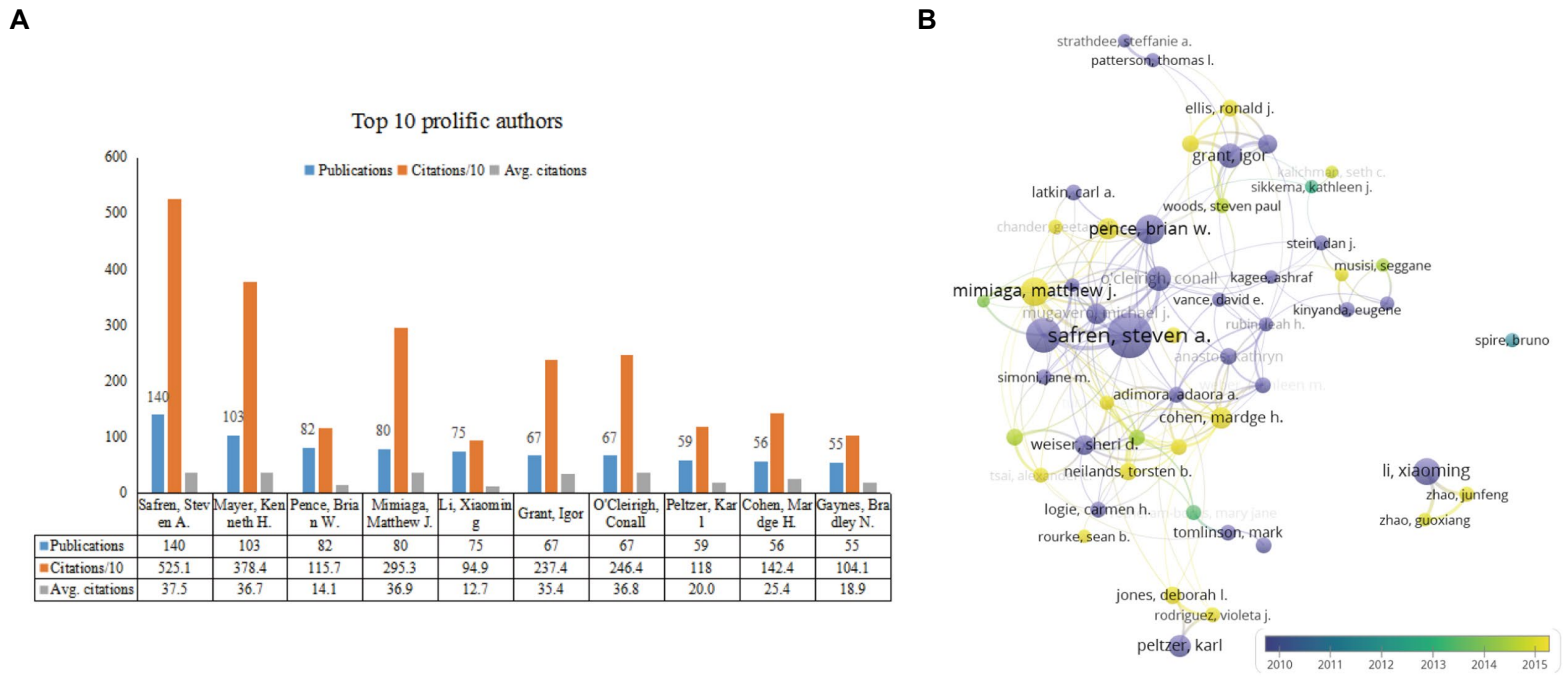
The top 10 prolific authors and inter-authors co-operation relationships on depression-related research in HIV/AIDS. **(A)** The number of publications, citations/10, and Avg citations in the top 10 authors. **(B)** The co-authorship network visualization map of the authors for depression-related research in HIV/AIDS. Node size indicated the number of articles produced. The distance between any two nodes positively associated with the cooperation strength. The color indicated the average publication year for the author. The blue color represented for early stage and yellow color represented late stage.

Figure 5 displays the top contributing authors (at least 30 papers) and collaborative map between them. Safren, Steven A. (University of Miami, United States) with 140 publications, Mayer, Kenneth H. (Fenway Health, United States) with 103 publications, and Pence, Brian W. (University of North Carolina, United States) with 82 publications were identified as the top three most prolific authors. In terms of citations, Safren, Steven A. also ranked first with 5251 TC and 37.5 CPP, followed by Mayer, Kenneth H. with 3784 TC and 36.7 CPP and Mimiaga, Matthew J. (University of California Los Angeles, United States) with 2953 TC and 36.9 CPP. We used VOSviewer to demonstrate the inter-authors cooperative network and their active time. Figure 5B displayed that a total of 53 authors have more than 30 publications in this filed. The largest cluster consists mainly of Safren, Steven A. Mimiaga, Matthew J., Pence, Brian W., and others, mainly from the United States. A cluster led by Peltzer, K, Jones, D L. Rodriguez, V J., who are primarily from South Africa and the United States, focuses on PLWH in South Africa. The cluster formed by Li xiaoming, Zhao guoxiang, Zhao junfeng, are all researchers from China. Additionally, we used a timeline view to present the active time of different authors in this field.

### 3.6. Top contributing journals

Table 1 presents the top 10 most prolific journals and co-cited journals. AIDS Care (*n* = 683 publications) was the top prolific journal, followed by AIDS Behavior (*n* = 615 publications) and JAIDS-J Acquired immune deficiency syndromes (*n* = 224 publications). However, in terms of CPP, AIDS (*n* = 153 publications) has recorded the highest CPP (*n* = 46.4 CPP), followed by JAIDS-J Acquired immune deficiency syndromes (*n* = 224 publications, 41.3 CPP) and AIDS Patient Care and STDS (*n* = 198 publications, 39.6 CPP). Co-cited journals were journals frequently cited in a series of selected publications. Of which, AIDS Behavior (*n* = 12,302 co-citations), AIDS Care (*n* = 11,426 co-citations), and AIDS (*n* = 9,775 co-citations) ranked the first three.

**Table 1:** Top 10 prolific journals and co-cited journals.

Rank	Journal	Publications	TC	CPP	IF (2021)	Cocited journal	Co-citations	IF (2021)
1	AIDS Care	683	13,935	20.4	1.89	Aids Behav	12,302	4.85
2	AIDS Behav	615	13,860	22.5	4.85	Aids Care	11,426	1.83
3	JAIDS-J Acq Imm Def	224	9,256	41.3	3.77	AIDS	9,775	4.63
4	Plos One	220	5,147	23.4	3.75	JAIDS-J Acq Imm Def	7,750	3.77
5	AIDS Patient Care St	198	7,838	39.6	5.94	Am J Public Health	5,771	11.56
6	AIDS	153	7,104	46.4	4.63	AIDS Patient Care St	5,748	5.94
7	J Assoc Nurse AIDS C	131	1903	14.5	1.81	Plos One	5,316	3.75
8	BMC Public Health	120	2,370	19.8	4.14	Lancet	4,906	202.73
9	Drug Alcohol Depen	86	2,227	25.9	4.85	Am J Psychiat	4,714	19.24
10	Int J Std AIDS	84	1,100	13.1	1.46	JAMA-J Am Med Assoc	4,703	157.33

### 3.7. Top cited articles

Citation is a quotation from one article to another article. The number of citations to the literature has been used as an indicator of the value, interest and utility of different articles in terms of the advancement of knowledge (González-Alcaide et al., 2016). Table 2 covered the top 20 most cited publications in this field. Among them, six publications are review, and the remaining publications are original articles. The most cited article (6677 TC) was published by Mathers, CD et al. in PLos Med., entitled “Projections of global mortality and burden of disease from 2002 to 2030” (Mathers and Loncar, 2006). In this article, the authors project disease-related mortality and major disease burden by 2030 based on model and baseline data. Specifically mentioned that HIV/AIDS become the leading disease burden by 2015 in middle- and low-income countries. Additionally, Seven of the top eight articles are studies on the global burden of disease, and all suggest that HIV/AIDS is a leading cause to the global burden of disease (Lopez et al., 2006; Mathers and Loncar, 2006; Murray et al., 2012; Vos et al., 2012; Global Burden of Disease Study, C, 2015; Disease et al., 2016; GBD 2019 Diseases and Injuries Collaborators, 2020).

**Table 2:** Top 20 most cited publications.

Rank	First author	Title	Document type	TC	Journal	Year
1	Mathers, CD	Projections of global mortality and burden of disease from 2002 to 2030	Article	6677	PLos Med.	2012
2	Murray, CJL	Disability-adjusted life years (DALYs) for 291 diseases and injuries in 21 regions, 1990–2010: a systematic analysis for the Global Burden of Disease Study 2010	Article	5440	Lancet	2006
3	Vos, T	Years lived with disability (YLDs) for 1,160 sequelae of 289 diseases and injuries 1990–2010: a systematic analysis for the Global Burden of Disease Study 2010	Article	4449	Lancet	2016
4	Lopez, AD	Global and regional burden of disease and risk factors, 2001: systematic analysis of population health data	Article	3831	Lancet	2015
5	Vos, T	Global, regional, and national incidence, prevalence, and years lived with disability for 310 diseases and injuries, 1990–2015: a systematic analysis for the Global Burden of Disease Study 2015	Article	3603	Lancet	2007
6	Vos, T	Global, regional, and national incidence, prevalence, and years lived with disability for 301 acute and chronic diseases and injuries in 188 countries, 1990–2013: a systematic analysis for the Global Burden of Disease Study 2013	Article	3451	Lancet	2020
7	Prince, M	Global mental health 1 – No health without mental health	Review	1876	Lancet	2004
8	Abbafati, C	Global burden of 369 diseases and injuries in 204 countries and territories, 1990–2019: a systematic analysis for the Global Burden of Disease Study 2019	Article	1476	Lancet	2011
9	DiMatteo, MR	Variations in patients’ adherence to medical recommendations – A quantitative review of 50 years of research	Review	1422	Med. Care	2015
10	De Hert, M	Physical illness in patients with severe mental disorders. I. Prevalence, impact of medications and disparities in health care	Article	1322	World Psychiatry	2003
11	Whiting, PF	Cannabinoids for Medical Use A Systematic Review and Meta-analysis	Review	1165	JAMA-J. Am. Med. Assoc.	2020
12	Burke, BL	The efficacy of motivational interviewing: A meta-analysis of controlled clinical trials	Article	973	J. Consult. Clin. Psychol.	2001
13	Rogers, JP	Psychiatric and neuropsychiatric presentations associated with severe coronavirus infections: a systematic review and meta-analysis with comparison to the COVID-19 pandemic	Review	932	Lancet Psychiatry	2001
14	Berger, BE	Measuring stigma in people with HIV: Psychometric assessment of the HIV stigma scale	Article	925	Res. Nurs. Health	2018
15	Bing, EG	Psychiatric disorders and drug use among human immunodeficiency virus-infected adults in the United States	Article	900	Arch. Gen. Psychiatry	2014
16	Ferrucci, L	Inflammageing: chronic inflammation in ageing, cardiovascular disease, and frailty	Review	843	Nat. Rev. Cardiol.	2011
17	Schmitt, MT	The Consequences of Perceived Discrimination for Psychological Well-Being: A Meta-Analytic Review	Article	843	Psychol. Bull.	2013
18	Walker, SP	Child Development 1 Inequality in early childhood: risk and protective factors for early child development	Article	824	Lancet	2008
19	Bockting, WO	Stigma, Mental Health, and Resilience in an Online Sample of the US Transgender Population	Article	816	Am. J. Public Health	2012
20	Chida, Y	Positive psychological well-being and mortality: A quantitative review of prospective observational studies	Review	813	Psychosom. Med.	2006

### 3.8. Analysis of co-citation references

**Figure 6:**
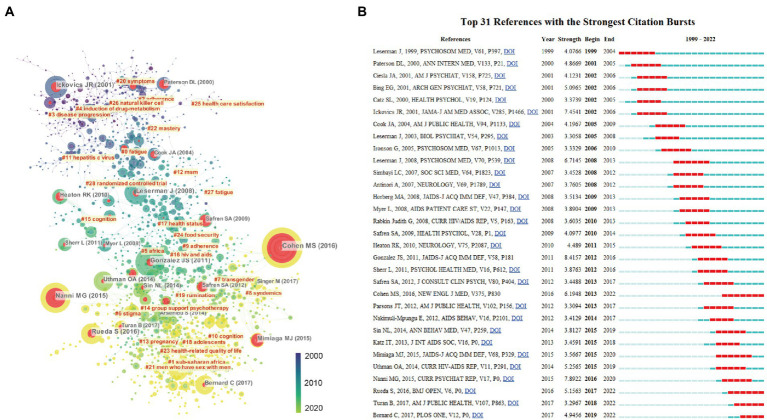
Analysis of co-citation references related to publications of depression on HIV/AIDS field. **(A)** The co-occurrence network visualization map of co-citation references related to depression in HIV/AIDS field. Large nodes indicate they received more citations, and the red nodes represent an outstanding contribution to this field’s development. The color indicated the publication year for the co-citation references. The blue color represented for early stage and yellow color represented late stage. **(B)** Top 31 references with the strongest citation bursts. The red lines represented their active time.

Co-citation is defined as two publications being cited by a third at the same time (González-Alcaide et al., 2016). The analysis of co-citations is the identification and quantification of the frequency of co-occurring literature in the reference list in a research field. This helps us to identify the most influential literature in the field. The co-citation analysis was performed with CiteSpace and VOSviewer to examine the evolution of scientific paradigms in depression-related research on HIV/AIDS field. Figure 6A showed the publications have more than 20 citations. Large nodes indicate they received more citations, the red nodes represent an outstanding contribution to this field’s development. They connected important references in sub-domains of this field. For example, “Leserman J, 2008, Psychosom Med, V70, P539, (Leserman, 2008)” entitled “Role of depression, stress, and trauma in HIV disease progression.” This article bridges the knowledge between the cluster# 16 (hiv/aids) and cluster# 27 (fatigue). Moreover, CiteSpace also identified the influential co-cited references in different time. Articles published around 2000 were centered on topics about “symptoms,” “disease progression,” and “induction of drug-metabolism.” Articles published around 2010 were centered on topics about “adherence,” “fatigue,” “Africa,” and “transgender.” Finally, articles published around 2020 were centered on topics about “stigma,” “group support psychotherapy,” “cognition,” and “MSM.” Figure 6B lists these important articles and their active time. Furthermore, we identified several articles received lots of attentions from 2020 to 2022, including Cohen MS, 2016, New Engl J Med, V375, P830, (Cohen et al., 2016), which found early initiation of ART led to a sustained decrease in genetically linked HIV-1 infections in sexual partners; Rueda S, 2016, BMJ Open, V6, P0 (Bernard et al., 2017), which meta-analyzed the prevalence and factors of depression among PLWH in sub-Saharan Africa; Turan B, 2017, AMJ Public Health, V107, P863 (Turan et al., 2017), which developed a conceptual framework of HIV-related stigma to explore the four ways in which HIV-related stigma differently affects the health of patients.

### 3.9. Analysis of keywords

**Figure 7:**
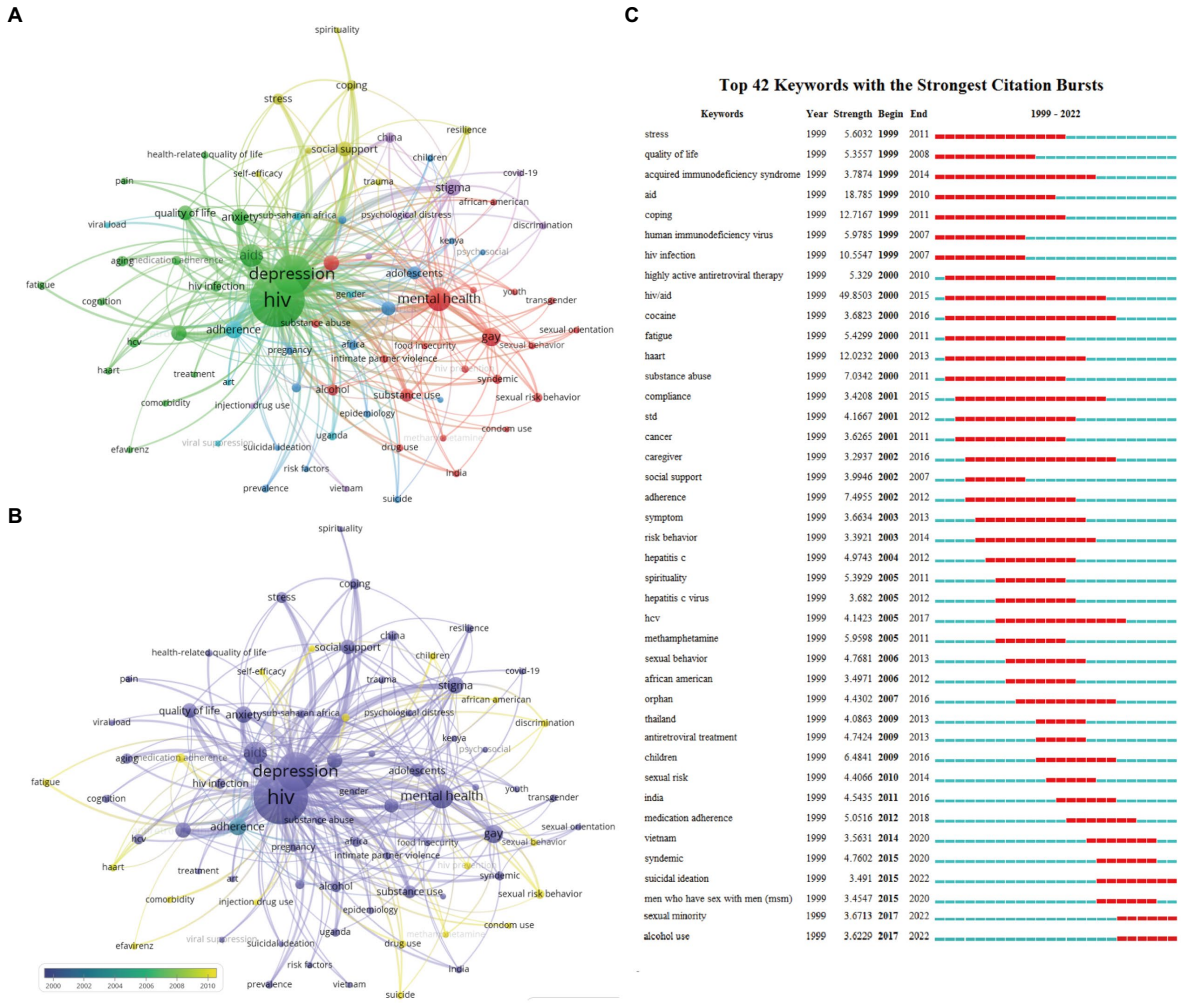
Analysis of keywords related to publications of depression on HIV/AIDS field. **(A)** The co-occurrence network visualization map of keywords related to depression in HIV/AIDS field. The keywords were clustered into five groups according to their color. Large nodes represented keywords with high frequency; **(B)** Keywords were colored according to the appearance for the average time. The blue color represented for early stage and the yellow color represented late stage. **(C)** The top 42 keywords with the strongest citation bursts.

VOSviewer and CiteSpace were used to conduct keyword co-occurrence analysis to present major hotspots and frontiers in this field. To count the number of keyword occurrences more accurately, we used a thesaurus (Supplementary Table S1) to merge keywords with similar meanings. For example, acquired immunodeficiency syndrome was replaced by aids, and human immunodeficiency virus was replaced by HIV. Finally, 83 keywords appeared more than 40 times were classified into five clusters (Figure 7A). The top three most occurred keywords are “HIV” (*n* = 2,703), “depression” (*n* = 1,605), and “mental health” (*n* = 641). Further, to decipher potential research frontiers in this field, we used the overlay map of keywords occurrence by VOSviewer and keyword citation burst by CiteSpace. Frequently used keywords can be detected during a particular period. Keyword citation burst is an increase in the frequency and number of keyword occurrences within a certain period of time (Li et al., 2020). Figure 7B displayed the potential hot keywords (yellow) identified by VOSviewer, including “medication adherence,” “suicide,” “discrimination,” “social support,” “self-efficient,” and so on.” Figure 7C identified the evolution of keywords from 1999 to 2022. The top 42 keywords with the strongest citation bursts are displayed, which show that “hiv/aid” is the keyword with the highest burst strength (*n* = 49.9). In the early stage, “stress,” “quality of life,” “cocaine” received lots of attention. And then keywords like “social support,” “adherence,” “risk behavior” got much attention. In recent years (2010-now), keywords have received more attention (e.g., “sexual risk behavior,” “medication adherence,” “suicide” and “condom use”), which suggests that these keywords are likely to become frontier hot topics in the future.

## 4. Discussion

### 4.1. Analysis of publications trend

In this study, 8,190 publications were included in the final analysis. We observed from Figure 2 that the trend of publications was slowly increasing from 1999 to 2021, which suggests that there is an increasing interest among scholars in different disciplines to focus on depression in HIV/AIDS-related research. It is worth noting that during the COVID-19, the number of publications remains high, with 697 publications in 2020 and 692 publications in 2021. Although the topics are still dominated by Public Environmental Occupational Health, Infectious Diseases, and Psychiatry in the last 3 years of publications, a portion of the studies have focused on the impact of pandemic outbreaks on PLWH. Many scholars became concerned about the impact of SARS-CoV-2 and lockdown on mental health of PLWH during COVID-19. First, patients with a deficient immune system will be more vulnerable to being affected by SARS-CoV-2 (Zhao et al., 2020), which aggravates their anxiety and worries in patients with AIDS. Second, the lockdown and social isolation increase the economic burden for HIV patients and their family members (Jones et al., 2021). Third, the COVID-19 pandemic caused extensive clinic pauses and high-contagious SARS-COV-2 may lower the patients’ visit rate to the hospital (López and Solis-Soto, 2022). Jamile Ballivian conducted a cross-sectional study in Argentina and found decline in medication adherence, disruption to mental health services and substance abuse treatment. During lockdown (Ballivian et al., 2020). Prior study evidenced that depression among HIV patients is associated with lower adherence of ART and health outcomes (Mayston et al., 2012). Therefore, pandemic outbreak is a huge challenge for public health, especially for special vulnerable populations like PLWH. The mental health of PLWH deserves focused attention during COVID-19.

### 4.2. Analysis of countries

Figures 3A,B showed that the United States dominated the field of AIDS-related depression, with the highest number of publications (68.6% of the total) and at the core of cooperation between countries/regions. The United States is at the center of the map and is connected to many countries, demonstrating the depth and breadth of the collaboration between U.S. researchers and South Africa, the United Kingdom, Canada, and China. Previous bibliometric analysis also reported that the United States plays the leading role in other areas of HIV (Liang et al., 2021), such as HIV dementia (Hussain et al., 2022), AIDS-related stigma and discrimination (Sweileh, 2019), and gerontology research in the field of HIV (Hoang et al., 2022), etc. South Africa ranked second in the number of publications. This result corresponds to the regional distribution of the PLWH. In 2021, 20.6 million people were living with HIV in Eastern and Southern Africa, nearly half of the total global PLWH (World Health Organization, 2021). Interestingly, when it comes to CPP, Australia ranked first with 44.3 CPP. A potential explanation for its high citations is that many of its’ publications published in *Lancet*, a very authoritative journal. The top most cited articles, all collaborations between the University Queensland, University Washington, and Harvard University, were published in *Lancet*. They analyzed the global burden of disease and mortality, which suggested that HIV/AIDS and depression were the top 10 leading diseases for global disease burden in recent decades (Lopez et al., 2006; Murray et al., 2012; Vos et al., 2012; Disease et al., 2016).

### 4.3. Analysis of institutions and authors

As shown in Figure 4A, all the top 10 prolific institutions were from the United States, corroborating the dominance of the United States in the field of HIV-related depression. Figure 4B illustrates the collaboration between the different institutions. Geographical distribution can affect the scientific cooperation between authors, institutions, and even countries (Baghaei Lakeh and Ghaffarzadegan, 2017). Researchers tend to choose the closer one as their collaboraters because of similar culture background. The institutions from the United States collaborate relatively more intensive and extensive. The cooperation of these institutions has significantly contributed to the number and impact of U.S. publications. Figure 4B also showed a very close collaboration between South Africa and the United States, mainly between Columbia University and University Cape Town. They conducted a cross-sectional study from October 2004 to December 2005, include 465 participants, to analyze the high prevalence of depression, anxiety, and substance-related disorders, and validate some brief rating scales which could be used to identify psychopathology in HIV-positive individuals (Myer et al., 2008).

Interestingly, although Harvard Univ was not one of the top three productive institutions, it ranked first regarding average citations (CPP = 68.3). We reasoned that this may be attributed to the top prolific author, Safren, SA (University of Miami, United States). He has established a close collaboration with Mayer, KH. and Mimiaga, MJ, and Pence, BW, all of them ranked the top 10 prolific authors in Figure 5A. They focused on depression and other psychological problems in HIV patients and revealed a significant positive association between depression and ART nonadherence in PLWH (Gonzalez et al., 2011; Uthman et al., 2014). Besides, Safren, SA and Mayer, KH focused on the effect of cognitive-behavioral therapy to enhance medication adherence and reduce depression among PLWH (Safren et al., 2009). Besides research team from the United States, a research team from China, represented by Li, Xiaoming also attracted our attention. They were very active in recent years (Figure 5B, colored yellow). They focused on the depression and social support among children affected by parental HIV in China (Ezell et al., 2020; Wu et al., 2022; Jiang Y. et al., 2022; Jiang Y. P. et al., 2022), the relationship between depression, HIV stigma and sexual risk factors among men who have sex with men (MSM) in China (Sun et al., 2020; Mi et al., 2022). Since the outbreak of the COVID-19, they also paid attention to the stress and coping strategies among HIV healthcare providers (Mi et al., 2021).

### 4.4. Analysis of keywords and research frontiers

Analysis of keywords can help to quickly understand the knowledge network of a field. As shown in Figures 7B,C, in the early stages, the main keywords include “cocaine,” and “quality of life.” Heaton et al. examined the relationship between neuropsychological deficits and quality of life in HIV-infected in 2004 (Heaton et al., 2004); Sadek et al. assessed the impact of neuropsychological deficits and depression on methamphetamine abuse and quality of life of PLWH in 2007 (Sadek et al., 2007); and Mindt et al. conducted a similar study in Spain in 2003 (Mindt et al., 2003). They provide clinical evidence for future research on HIV-associated neuropsychiatric deficits. In the last few years, “sexual minority,” “alcohol abuse,” and “suicidal ideation” have attracted the attention. PLWH are at high risk for suicidal ideation and behavior. In order to reduce the risk of suicide among PLWH, many scholars have conducted research on the protection and risk factors of suicidal ideation. Casale found that stigma is a highly significant risk factor among adolescents living with HIV, and social support can buffer the adverse effects of stigma (Casale et al., 2019). Wang et al. demonstrated, in the form of a structural equation model, that stigma is the most influential factor in the accumulation of suicidal ideation among AIDS patients in China, while self-esteem and depression are also important risk factors for suicidal ideation (Wang et al., 2018). They all agree that improving tangible social support for PLWH can mitigate the risk of suicidal ideation in future research.

In this study, we visualized the knowledge networks and research paradigms of depression-related research among HIV/AIDS. The results showed 83 keywords appeared more than 40 times in Figure 7A. “HIV” and “depression” are the most frequently occurring keywords. The six largest clusters mentioned below represent the whole study field.

#### 4.4.1. Cluster 1 focus on the ART and adherence

The primary keywords are “adherence (*n* = 346),” “antiretroviral therapy (ART) (*n* = 322),” “medication adherence (*n* = 101),” “highly active antiretroviral therapy (HARRT) (*n* = 94),” and “cognition (*n* = 81).” Since the inception of ART, the lifespan of HIV patients has been greatly improved. Adherence to ART plays a significant role in the effectiveness of treatment (Chesney, 2003). Especially, previous study produced by Cohen et al. evidenced that ART can effectively prevent the transmission of HIV and subsequently reduce the morbidity of HIV/AIDS (Cohen et al., 2016). As shown in Figure 7B, highly active antiretroviral therapy (HARRT, cocktail therapy) has been a hot topic in recent years. Compared with traditional single antiviral drug, HARRT combines different categories’ antiviral chemicals and prolongs the HIV/AIDS patient survival (~7–10 years; Lu et al., 2018). Nevertheless, the complexity of regimens, high economic cost and frequent side effects (e.g., gastrointestinal reactions depression, insomnia, headaches) make HAART particularly difficult to insist (Trotta et al., 2002; Husstedt et al., 2009; Endalamaw et al., 2018). Cruess et al. (2012) demonstrated depressive symptoms were associated with nonadherence of antiretroviral and psychotropic medication (Cruess et al., 2012). Similar to Cruess’ finding, a meta- analysis also found that lower adherence can impair mental health of PLWH (Gonzalez et al., 2011). Using cognitive behavioral therapy to improve adherence and depression have proven to be effective among PLWH in recent years. For example, Safren et al. (2021) validated the task-shared cognitive-behavioral therapy can increase the rate of adherence (~0.13 percentage points) and improve depression scores (estimated 4.88 points *via* Hamilton Depression Scale) in PLWH in Khayelitsha, South Africa (Safren et al., 2021). Han, et al. (2021) tested the effects of nursing leading cognitive behavioral intervention on depression, anxiety, and ART medication adherence in PLWH in China, and found that a greater improvement was shown in primary outcome after the intervention, and yet no positive effect was found after 6 months of follow-up (Han et al., 2020). However, contrary to the previous studies, Donenberg’ team (2022) compared Trauma-Informed Cognitive Behavioral Therapy enhanced to address HIV (TI-CBTe) with usual care in adolescents living with HIV. They suggested that there was no significant advantage of TI-CBTe in improving adherence. In conclusion, depression negatively affect medication adherence and thus affect the health outcome of HIV patients. Further research on interventions to improve depression and adherence needs to be explored.

#### 4.4.2. Cluster 2 focus on the HIV-related depression in men has sex with men (MSM)

The primary keywords are “MSM (*n* = 327),” “sexual risk behavior (*n* = 45),” “sexual orientation (*n* = 61),” “condom use (*n* = 47)” and “childhood sexual abuse (CSA) (*n* = 31).” MSM remain the high-risk group for HIV, accounting for 69% of new HIV-positive diagnoses in the United States in 2020 (World Health Organization, 2022a). The stigmatization of the sexual orientation has never ended among HIV-positive MSM (Turan et al., 2019), since the disease was first documented as a “gay-related immunodeficiency” (Epstein, 1997). Studies in both developed and developing countries have reported high levels of stigma in MSM, such as the United States (Quinn et al., 2017; Brewer et al., 2020; Babel et al., 2021), China (Wu et al., 2015; Yang et al., 2020), Nigeria (Rodriguez-Hart et al., 2017), Vietnam (Chen et al., 2022), etc. Moreover, MSM who experienced stigma have a high risk of depression and high-risk sexual behaviors while health care utilization was low (Chen et al., 2015). As shown in Figure 7B, “sexual risk behavior” and “condom use” are the more popular keywords in the recent decade. Many scholars have devoted their efforts to exploring the relationship between depression and sexual risk behavior in MSM (Stall et al., 2003; Koblin et al., 2006). For example, in 2012, Blashill et al. recruited 430 HIV-positive MSM from the largest HIV outpatient in Boston and evaluated whether body mass index (BMI) is associated with serodiscordant unprotected anal intercourse (SDUAI). The authors reported that HIV-positive MSM with underweight and depression may be more likely to occur SDUIA (Blashill et al., 2012). Alvy et al. (2011) evidenced that self-efficacy and cognitive escape mediated the relationship between depression and high-risk sexual behavior (Alvy et al., 2011). Besides, CSA also gained a lot of attention in recent decades. Previous studies reported a very high percentage of CSA among MSM, estimated from 20% to 40% (Mimiaga et al., 2009; Williams et al., 2015; Tomori et al., 2016). Importantly, as early as 2001, Paul et al. reported that CSA will lead to depressive and self-destructive tendencies in MSM group, which in turn predispose such men to engage in HIV-related risky sexual behavior (Paul et al., 2001). Consistent with the finding of Paul et al., Mimiaga et al. (2009) indicate that HIV-uninfected MSM with a history of CSA may have a greater risk of HIV infection and a higher rate of HIV sexual risk behaviors (Mimiaga et al., 2009). Thus, we call for an acute need for early identification of CSA risk factors and propose that health interventions targeting MSM who have experienced CSA should be added to future HIV prevention studies.

#### 4.4.3. Cluster 3 focus on the impact of stigma on PLWH

The primary keywords are “stigma (*n* = 328),” “social support (*n* = 266),” “discrimination (*n* = 63),” “China (*n* = 132),” and “COVID-19 (*n* = 61).” Stigma and discrimination are one of the biggest issues facing PLWH (World Health Organization and Unaids, 2022). Many studies develop measurement scales to evaluate HIV-related stigma (HRS). Berger et al. (2001) first developed and validated the HIV Stigma Scale (HSS, including 40 items) from the perspective of psychosocial aspects. The authors found that HRS is significantly associated with depression (Berger et al., 2001). This paper is also one of the top 20 most cited publications (Table 2). To widely applied in clinic practice and without compromise its’s reliability and validity, Wright et al. (2007) revised the HSS to a shorter 10-item scale (Wright et al., 2007). After being cross-culturally adapted several times, HSS has applied in many countries and regions, such as Sweden (Wiklander et al., 2013), Spanish-speaking culture (Jimenez et al., 2010), China (Yu et al., 2019), Brazilian (Luz et al., 2020), Japan (Kagiura et al., 2020), and so on. In addition to the development of scales to examine stigma among PLWH, several studies have also examined the effect of social support on stigma and depression. Previous studies have reported stigma is positive associated with depression (Murphy et al., 2006; Kota et al., 2022; Stojanovski et al., 2022). A systematic review also reported PLWH living with HRS was 1.6 times more likely to suffer depression. And interestingly, the authors also found that high levels of social support may decrease the negative effects (anxiety, depression) of HRS on mental health (Armoon et al., 2022). This is likely due to social support can improve self-efficacy and help patients to seek timely medical care (Turan et al., 2016). During COVID-19, PLWH may report more types of discrimination (Tomar et al., 2021). A systematic review published in *Lancet Psychiatry* in 2020 drew public attention to psychiatric and neuropsychiatric related to the COVID-19 pandemic (Rogers et al., 2020). The authors suggested that SARS-CoV-2 may cause depression, anxiety, fatigue, and post-traumatic stress disorder in patients because of the continuous COVID-19 pandemic and the subsequent policy of lockdown and social isolation (Marbaniang et al., 2020). Li et al. compared COVID-19 Stigma and HRS in China (Li et al., 2021). The author found that the two both include isolation and avoidance of the patients, but COVID-19 is the less moral condemnation, while PLWH are easier to be discriminated. Stigma against infectious diseases (including COVID-19 and HIV) need to be reduced to improve the mental health of patients. We call for more social support to reduce HRS and alleviate depression in the future.

#### 4.4.4. Cluster 4 focus on substance abuse associated with HIV-related depression

The primary keywords are “substance abuse (*n* = 278),” “alcohol use (*n* = 149),” “drug use (*n* = 70),” and “methamphetamine (*n* = 55).” Approximately 14% of the global burden of disease is caused by neuropsychiatric diseases, of which, depression, alcohol use, and substance use are the top three reasons (Prince et al., 2007). Injecting drug use has been one of the major methods of HIV transmission. Drug addiction (such as cocaine cannabis, and marijuana) and AIDS are entwined diseases. Previous studies have found a high prevalence of psychiatric disorders, substance use, and drug abuse among PLWH. A national survey including 2,864 HIV/AIDS in the United States reported that nearly half of the participants suffered from mental disorders and drug use (Bing et al., 2001). A cross-sectional study in the Asia-Pacific region found 80% of PLWH had substance use, and 48% had depression (mild-to-severe depression; Ross et al., 2022). Literatures suggested that cocaine has adverse effects on HIV-associated neurocognitive disorder and neuro AIDS (Dahal et al., 2015; Illenberger et al., 2020). Additionally, several systematic reviews reported that substance abuse and depression are high risk factors for suicide in PLWH (Metekiya et al., 2022; Tsai et al., 2022). More research on the role of drug abuse (include cocaine, cannabis, and marijuana) in HIV infected individuals could contribute to the development of novel treatment strategies for PLWH with substance abuse. In recent years, many studies have focused on the intersection of alcohol abuse and depression in PLWH. Madkour and Felker-Kantor found the PLWH with higher alcohol consumption showed worse depression and anxiety (Madkour et al., 2022). Kalichman reported that the combination of depression and alcohol use have detrimental effects on ART adherence and subsequently result in virologic failure and poor treatment outcomes (Sinclair et al., 2014; Soboka et al., 2014; Kalichman et al., 2022). This means that alcohol abuse not only leads to worse depression but also negatively affects the health outcome of PLWH. Caniglia et al. proposed that screening and treatment for alcohol and depression may help alleviate comorbidity of anxiety, other substance abuse, etc. in PLWH (Caniglia et al., 2021). It’s time to incorporate screening and detox for alcohol, drugs, and cocaine into routine clinical care for HIV, especially the management of alcohol in men (Lancaster et al., 2021; Maisto et al., 2021). Future research should encourage the establishment of an integrated screening and treatment program.

#### 4.4.5. Cluster 5 focus on mental health

The primary keywords are “anxiety (*n* = 315),” “quality of life (*n* = 258),” “stress (*n* = 141),” and “suicide (*n* = 68).”There has been substantial evidence that the depression and anxiety experienced by HIV-infected patients are related to neurobiological changes caused by the ongoing attack on the central nervous system by the virus (Kopnisky et al., 2007). Over the past 20 years, many studies focused on mental disorders and quality of life in PLWH. The most common neuropsychiatric complication among PLWH is depression, which also greatly limits the quality of life of patients (Nanni et al., 2015). Angelino and Treisman have outlined the diagnosis and treatment of psychiatric disorders such as depression, bipolar disorder, and substance abuse that occur in PLWH, and argued that treatment of these disorders can greatly improve health outcomes in patients (Angelino and Treisman, 2001). Whetten et al. suggested that negative experiences such as trauma, mental disorders and stigmatization are strongly associated with increased risk behaviors and decreased quality of life in HIV patients (Whetten et al., 2008). These factors are critical to HIV treatment and prevention. We recommend the establishment of referral mental health service centers based on community health care systems to meet the mental health needs of HIV-positive patients. The mental health issues that can arise in older adults with AIDS become critical as the proportion of older cases increases (Brooks et al., 2012). Older HIV-positive patients are known to suffer from poorer cognitive function and have a higher risk of depression compared to the general population (Mitra et al., 2022). Some experts suggest that this is related to cumulative AIDS-related medical and psychiatric stress, as well as the immune deficiency caused by HIV (Rodriguez-Penney et al., 2013). Yoo-Jeong et al. found the role of loneliness as a mediator in the relationship between stigma and depressive symptoms (Yoo-Jeong et al., 2022). Studies have shown that reducing HIV stigma and loneliness in older HIV-positive patients can reduce depressive symptoms and improve quality of life (Grov et al., 2010). Overall, our recommendation is that further explanation of loneliness may have a lasting impact on understanding mental health and improving depressive symptoms in older adults living with HIV.

#### 4.4.6. Cluster 6 focus on research in sub-Saharan Africa

The primary keywords are “South Africa (*n* = 231),” “women (*n* = 283),” “pregnancy (*n* = 88),” “adolescents (*n* = 56),” “uganda (*n* = 94),” and “Kenya (*n* = 60).” Under this cluster, they focus on adolescents and women living with HIV. Globally, there are 1.6 million adolescents living with HIV, 70% of whom are living in Eastern and Southern Africa (World Health Organization, 2022b). Correspondingly, most research has focused on Eastern and Southern Africa. A cross-sectional study in Botswana, which ranked among the top four countries most affected by HIV, reported the prevalence of depression among adolescents living with HIV were 23% (Olashore et al., 2022), which is consistent with the rate (24–27%) in the systematic review in sub-Saharan (Dessauvagie et al., 2020). West et al. (2019) found that the majority of HIV-infected adolescents were infected perinatally, and the adolescents who receive earlier and higher social support were less likely to have mental health symptoms (West et al., 2019). That is to say, sub-Saharan African countries may not be able to provide appropriate and adequate mental health care for adolescents due to the high-burden of HIV/AIDS (Petersen et al., 2010). Adolescent psychological problems are also accompanied by suicidal behavior. It is reported that Africa has the highest prevalence of suicide behavior in adolescents worldwide, and the prevalence of suicidal ideation and suicide planning were 20.4% and 23.7%, respectively (Uddin et al., 2019). This group urgently needs more social support and attention in low-resource settings (Dow et al., 2016). In addition, the studies of women should not be underestimated. Peltzer et al. (2018) assess the postnatal depression among HIV-positive women in South Africa and reported both prenatal (20.1%) and postnatal (14.6%) depression have high prevalence (Peltzer et al., 2018). The main risk factors for depression in women during pregnancy include less education, intimate partner violence, lack of participation by men and lower adherence to ART (Peltzer et al., 2016, 2018; Rodriguez et al., 2018). Adolescents pregnancy is also a serious issue among women living with HIV. Millar et al. conduct a retrospective cohort (between January 1, 2005 and February 28, 2017) in western Kenya. 8,565 HIV-positive adolescents girls were included in this cohort and they found ~18.5% were pregnant <20 years of age (Millar et al., 2020). What’s more worried is that Sub-Saharan Africa has the highest rate of adolescents pregnancy in the world, almost 20% (Kassa et al., 2018). However, Roberts focus on the mental health among this group and suggest that there is an absence of evidence-based policy and programming for this group. Therefore, aim to eliminate the limitations imposed by regional and different cultural backgrounds, they need a multi-institution, multi-national academic research model to help alleviate HIV pressure in the African region, especially in adolescent and women.

### 4.5. Limitations

This study has several limitations that should be acknowledged. First, all the literatures in this bibliometric analysis were collected from the WOSCC, which only represents one aspect of this topic. Restricted by data format for bibliometric tools, the publications in other databases (Pubmed, Scopus, Google Scholar etc.) were not included in this study. Second, the indicators for the quality of an article, an author, or an institution only included the number of publications, TC, and CPP. Other metrics are widely used in other studies, such as H-index, Impact Factor, CiteScore, etc. Third, due to the included criteria of language and publication types, only articles or reviews in English were included in this analysis. However,“English” remains the most common language used in published academic literatures worldwide, future research should integrate multiple non-English language (e.g., Chinese, German) publications to provide a more comprehensive analysis of this field.

## 5. Conclusion

This bibliometric analysis provides an overview of depression-related research on HIV/AIDS from 1999 to 2022. This study reported the publication trend, major contributing countries/regions, authors, institutions, journals and mapped the knowledge network of depression-related research on HIV/AIDS. In this field, topics such as “adherence,” “mental health,” “substance abuse,” “stigma,” “men who have sex with men” and “south africa” have attracted considerable attention. Further work should emphasize the following initiatives: (1) strengthen the co-operation between authors, institutions, and countries. (2) exploring different interventions to improve adherence. (3) developing more organizations to help PLWH reduce stigma and provide social support, especially in MSM (4) increasing investments to low-resource settings, especially in Sub-Saharan Africa.

## Data availability statement

The original contributions presented in the study are included in the article/Supplementary material, further inquiries can be directed to the corresponding authors.

## Author contributions

XD and QZ conceived of the study, participated in its design, and drafted the manuscript. JL and JH involved in study design, obtained data and contributed to interpretation, and helped to draft the manuscript. JC provided the theoretical frameworks and performed much of the editing of the manuscript and provided the fund support. All authors contributed to the article and approved the submitted version.

## Funding

This study was supported by the Hunan Science and Technology Innovation Platform and Talent Plan (grant: 2017TP1004).

## Conflict of interest

The authors declare that the research was conducted in the absence of any commercial or financial relationships that could be construed as a potential conflict of interest.

## Publisher’s note

All claims expressed in this article are solely those of the authors and do not necessarily represent those of their affiliated organizations, or those of the publisher, the editors and the reviewers. Any product that may be evaluated in this article, or claim that may be made by its manufacturer, is not guaranteed or endorsed by the publisher.
