# Supplementary material for: Global trends in depression among patients living with HIV: A bibliometric analysis
Source: Front Psychol. 2023 Mar 9;14:1125300. doi: 10.3389/fpsyg.2023.1125300 (PMC10036061; doi:10.3389/fpsyg.2023.1125300)
Supplement: Supplementary file 1 [file Table_1.DOCX]

Table S1. Thesaurus

| label | replace by |
| --- | --- |
| acquired immunodeficiency syndrome | aids |
| adolescent | adolescents |
| alcohol use | alcohol |
| depressive symptoms | depression |
| gay men | gay |
| hepatitis c virus | hcv |
| hepatitis c | hcv |
| hiv/aids | hiv |
| hiv-1 | hiv |
| human immunodeficiency virus | hiv |
| major depressive disorder | depression |
| men who have sex with men | gay |
| mental illness | mental health |
| msm | gay |
| syndemics | syndemic |
| sexual risk | sexual risk behavior |
| meta-analysis | systematic review |
